# Assessing the Relationship Between Neighborhood Socioeconomic Disadvantage and Telemedicine Use Among Patients With Breast Cancer and Examining Differential Provisions of Oncology Services Between Telehealth and In-Person Visits: Quantitative Study

**DOI:** 10.2196/55438

**Published:** 2024-07-18

**Authors:** Jincong Q Freeman, Fangyuan Zhao, Frederick M Howard, Rita Nanda, Olufunmilayo I Olopade, Dezheng Huo

**Affiliations:** 1 Department of Public Health Sciences University of Chicago Chicago, IL United States; 2 Center for Health and the Social Sciences University of Chicago Chicago, IL United States; 3 Cancer Prevention and Control Program UChicago Medicine Comprehensive Cancer Center Chicago, IL United States; 4 Section of Hematology/Oncology, Department of Medicine University of Chicago Chicago, IL United States; 5 Center for Clinical Cancer Genetics and Global Health University of Chicago Chicago, IL United States

**Keywords:** telemedicine, telehealth equity, Area Deprivation Index, neighborhood socioeconomic disadvantage, disparities, oncology services, treatment consultation, genetic counseling, in-person visits, breast cancer, mobile phone

## Abstract

**Background:**

Since the COVID-19 pandemic began, we have seen rapid growth in telemedicine use. However, telehealth care and services are not equally distributed, and not all patients with breast cancer have equal access across US regions. There are notable gaps in existing literature regarding the influence of neighborhood-level socioeconomic status on telemedicine use in patients with breast cancer and oncology services offered through telehealth versus in-person visits.

**Objective:**

We assessed the relationship between neighborhood socioeconomic disadvantage and telemedicine use among patients with breast cancer and examined differential provisions of oncology services between telehealth and in-person visits.

**Methods:**

Neighborhood socioeconomic disadvantage was measured using the Area Deprivation Index (ADI), with higher scores indicating greater disadvantages. Telemedicine and in-person visits were defined as having had a telehealth and in-person visit with a provider, respectively, in the past 12 months. Multivariable logistic regression was performed to examine the association between ADI and telemedicine use. The McNemar test was used to assess match-paired data on types of oncology services comparing telehealth and in-person visits.

**Results:**

The mean age of the patients with breast cancer (n=1163) was 61.8 (SD 12.0) years; 4.58% (52/1161) identified as Asian, 19.72% (229/1161) as Black, 3.01% (35/1161) as Hispanic, and 72.78% (845/1161) as White. Overall, 35.96% (416/1157) had a telemedicine visit in the past 12 months. Of these patients, 65% (266/409) had a videoconference visit only, 22.7% (93/409) had a telephone visit only, and 12.2% (50/409) had visits by both videoconference and telephone. Higher ADI scores were associated with a lower likelihood of telemedicine use (adjusted odds ratio [AOR] 0.89, 95% CI 0.82-0.97). Black (AOR 2.38, 95% CI 1.41-4.00) and Hispanic (AOR 2.65, 95% CI 1.07-6.58) patients had greater odds of telemedicine use than White patients. Compared to patients with high school or less education, those with an associate’s degree (AOR 2.67, 95% CI 1.33-5.35), a bachelor’s degree (AOR 2.75, 95% CI 1.38-5.48), or a graduate or professional degree (AOR 2.57, 95% CI 1.31-5.04) had higher odds of telemedicine use in the past 12 months. There were no significant differences in providing treatment consultation (45/405, 11.1% vs 55/405, 13.6%; *P*=.32) or cancer genetic counseling (11/405, 2.7% vs 19/405, 4.7%; *P*=.14) between telehealth and in-person visits. Of the telemedicine users, 95.8% (390/407) reported being somewhat to extremely satisfied, and 61.8% (254/411) were likely or very likely to continue using telemedicine.

**Conclusions:**

In this study of a multiethnic cohort of patients with breast cancer, our findings suggest that neighborhood-level socioeconomic disparities exist in telemedicine use and that telehealth visits could be used to provide treatment consultation and cancer genetic counseling. Oncology programs should address these disparities and needs to improve care delivery and achieve telehealth equity for their patient populations.

## Introduction

### Background

In the United States, telemedicine use has risen over the years. According to the 2019 American Hospital Association annual survey, the percentage of telehealth programs implemented across hospitals increased from 43.1% in 2015 to 61.2% in 2017 [1]. Since the COVID-19 pandemic began, we have seen rapid and unprecedented growth in the demand for, and use of, telemedicine. A recent report from the Centers for Disease Control and Prevention has documented that the frequency of telehealth visits increased by 50% from 2019 to 2020 [2]. The increase in the use of telemedicine is also observed in populations of patients with cancer; for example, several studies conducted during 2020 and 2021 estimated that the prevalence of telemedicine use ranges from 34.9% to 64.9% among patients with breast or other cancers [3-11]. In 2020, the Centers for Medicare & Medicaid Services introduced policies that offered regulatory waivers and flexible reimbursement to Medicaid and Medicare providers for telehealth, contributing in part to the observed increase in telemedicine use and implementation [12,13]. In 2021, the American Society of Clinical Oncology performed a systematic literature review on telemedicine and published standards and recommendations for telehealth services and practices in the oncology setting [14]. Telemedicine helps facilitate access to health care and services for patients with cancer and their caregivers or family members. However, telehealth care and services are not equally distributed, and not all patients with cancer have equal access to telehealth care and services across different US regions. There are notable gaps in existing literature regarding the influence of neighborhood-level socioeconomic status (SES) on telemedicine use in patients with breast cancer and oncology services offered through telehealth versus in-person visits.

Neighborhood-level SES is a fundamental component of the social determinants of health framework [15,16]. Neighborhood socioeconomic disadvantage has been shown to negatively affect health outcomes [17,18], access to care and preventive services [19,20], survival outcomes [18,21], and quality of life [22] among patients with cancer [23]. Previous investigations have also found neighborhood socioeconomic disadvantage to be associated with a lower likelihood of telemedicine use among patients in primary care and hematology or oncology clinics, as well as among outpatients [8,24-28]. A study of 627 patients with cancer experiencing financial distress during the COVID-19 pandemic reported a 3% decrease in the rate of telemedicine use per 10-unit increase in the Area Deprivation Index (ADI) [8], a validated composite measure of neighborhood-level SES [29,30]. Fassas et al [27] conducted a univariate analysis of 64 patients with head and neck cancer, revealing no significant differential interest in telehealth visits based on the ADI. Another study noted a higher percentage of telehealth visits among patients residing in the least socioeconomically deprived neighborhoods (54%) than those in the most deprived neighborhoods (46.1%) in a large cohort of patients with hematologic malignancies and patients with cancer from Kaiser Permanente [28]. These prior studies either lacked significant sample sizes or included heterogeneous populations of patients with cancer. Therefore, these findings may not be generalizable to the population of patients with breast cancer.

In addition, whether provisions or receipts of oncology services differ between telemedicine and in-person office visits among patients with breast cancer is unclear. A recent retrospective analysis of 311 patients with cancer indicated that clinical practices, such as molecular test ordering and palliative care referrals, conducted through telehealth visits achieve similar efficiency to in-person visits [31]. A pilot study of 45 patients with advanced cancer in Mexico has suggested the feasibility of supportive care delivery via telemedicine [32]. Multiple studies have found telehealth provisions or visits to be feasible, effective, and safe for patient follow-ups after ambulatory or breast surgeries [33-36]. Earlier research has also demonstrated that, when comparing telehealth to in-person visits, patients with cancer in the United States, Canada, and Europe reported similar communication experiences or satisfaction with the oncology care consultations they received [7,37,38]. Moreover, telemedicine-based cancer genetic counseling has been shown to be feasible and effective and to achieve high degrees of satisfaction among providers as well as patients with colorectal, breast, or gynecologic cancer residing in remote or rural areas [39-42]. Although prior studies have elucidated the successful implementation of telemedicine and shown that certain types of cancer care and services delivered through telemedicine are equivalent to in-person office settings in mixed cohorts of patients with cancer, the results may not be applicable to patients with breast cancer. Furthermore, most of these studies were not able to examine the distributions of oncology services comparing telehealth and in-person visits because of small sample sizes and therefore are primarily descriptive. Understanding these associations can help oncology programs identify telehealth disparities and patient’s unmet needs, improve telemedicine practice and care delivery, reduce health disparities and inequities, and provide optimal support to patients with breast cancer.

### Objectives

To fill these critical gaps in the literature, we undertook this study primarily seeking to evaluate (1) the association between neighborhood socioeconomic disadvantage and telemedicine use and (2) the differences in provisions of oncology services comparing telehealth and in-person office visits. The secondary objectives of this study were to describe (1) common perceived challenges or concerns related to telehealth visits and (2) patient satisfaction with oncology services delivered via telemedicine in this cohort of patients with breast cancer.

## Methods

### Study Design and Population

This study used a cross-sectional design and analyzed data from patients with breast cancer enrolled in the ongoing Chicago Multiethnic Epidemiologic Breast Cancer Cohort (ChiMEC), which is a hospital-based cohort established at the University of Chicago Medicine in 1993 [43]. From July to September 2022, a total of 1868 questionnaires were sent to ChiMEC participants who consented to follow-up surveys, of whom 1236 (66.17%) responded. The study survey is provided in Multimedia Appendix 1. For this analysis, of the 1236 respondents, we included 1163 (94.09%) patients who reported having had either telemedicine or in-person visits in the past 12 months.

### Ethical Considerations

The University of Chicago Institutional Review Board reviewed and approved this study (approval 16352A). All participants provided written informed consent before taking part in the ChiMEC study and follow-up surveys.

### Measures

Neighborhood socioeconomic disadvantage was defined by the ADI, a composite measurement of patients’ neighborhood-level income, education, employment, and housing quality based on linked zip codes and US Census block groups [29,30]. For this study, participants’ residential addresses were geocoded to census block groups and then linked with the corresponding ADI national ranking percentile, which ranks neighborhoods by socioeconomic disadvantage at the national level in the United States. ADI scores range from 1 to 100, with higher scores reflecting higher levels of neighborhood socioeconomic deprivation. We further categorized ADI scores into quartiles. The first quartile represented the least socioeconomically deprived neighborhoods, whereas the fourth quartile represented the most deprived neighborhoods.

Telemedicine use was defined as having had a telehealth visit with a physician or other health providers in any specialty in the past 12 months and dichotomized as *yes* or *no*. For patients who used telemedicine, we asked whether their visits were conducted through telephone, videoconferencing, or both. Similarly, in-person visits were assessed by asking participants whether they had had an in-person office visit with a physician or other health providers in the past 12 months. Furthermore, participants were asked whether their telemedicine or in-person visits were related to 6 different types of oncology services: treatment consultation; review of laboratory, screening, and pathology test results; management of cancer symptoms and treatment side effects; cancer genetic counseling; cancer clinical trial follow-up; and informed consent for a cancer clinical trial. Common cancer symptoms and treatment side effects discussed during telehealth or in-person visits were also assessed, including hot flashes; chemotherapy-induced neuropathy, nausea, and vomiting; pain related to cancer or cancer treatment; depressive symptoms or mood changes; fatigue or tiredness; anxiety or stress; lymphedema; and insomnia or sleep problems.

In addition, we asked participants to report any challenges or concerns when using telemedicine, such as technology difficulty or lack of comfort with technology, lack of electronic device (eg, desktop computer, laptop computer, smartphone, or iPad), lack of high-speed internet or slow internet connection at home, compromised patient-provider communication, compromised patient-provider relationship, telemedicine not being offered at the clinic or by a provider, cost, and telemedicine not being covered by health insurance. We then asked how satisfied participants were with their telehealth or in-person visits, using a 5-point Likert scale (ie, *not at all*, *a little*, *somewhat*, *very*, and *extremely satisfied*). Participants were also asked how likely they were to continue using telemedicine, using another 5-point Likert scale (ie, *very unlikely*, *unlikely*, *neutral*, *likely*, and *very likely*).

Individual-level sociodemographic and clinicopathologic characteristics included age at survey, race, ethnicity, highest level of education, marital status, type of health insurance coverage, duration from cancer diagnosis to survey, Charlson comorbidity index (excluding breast cancer diagnoses), histologic type, American Joint Committee on Cancer stage group, molecular subtype, tumor grade, receipt of cancer treatment (chemotherapy, hormone therapy, or radiotherapy), and type of surgery. We obtained patients’ clinicopathologic information from electronic health records and the hospital cancer registry. Distance from residence to hospital (in miles) was geocoded and calculated by taking the differences of coordinates (longitudes or latitudes) between the patient’s address and the University of Chicago Medicine Comprehensive Cancer Center’s address based on the Haversine formula.

### Statistical Analysis

We described patients’ characteristics using summary statistics. Means and SDs or medians and IQRs were calculated for continuous variables, and we used 2-tailed *t* tests, Wilcoxon rank sum tests, or Kruskal-Wallis tests to conduct bivariate analyses. For nominal data, we tabulated frequencies and percentages and compared the distributions using Pearson chi-square or Fisher exact tests. To examine the association between neighborhood socioeconomic disadvantage (continuous ADI scores) and telemedicine use, we fitted 3 separate multivariable logistic regression models. For modeling, we implemented a stepwise regression approach. Potential confounders were selected and adjusted for in the models based on a *P* value of <.10 from bivariate analyses or a priori knowledge. Model 1 included ADI, age at survey, race, ethnicity, duration from cancer diagnosis to survey, highest level of education, marital status, type of health insurance coverage, Charlson comorbidity index, and distance from residence to hospital. Model 2 was controlled for histologic type, American Joint Committee on Cancer stage, molecular subtype, and tumor grade, in addition to the covariates in model 1. Model 3 contained all variables in model 2 plus receipt of chemotherapy, hormone therapy, or radiotherapy, as well as type of surgery. Adjusted odds ratios (AORs) and corresponding 95% CIs were calculated. To evaluate the differences in types of oncology services between telemedicine and in-person office visits, we conducted McNemar tests on match-paired data of patients having both visit modalities. *P* values (2-tailed) <.05 were considered statistically significant. All statistical analyses were performed using Stata 17 (StataCorp LLC).

## Results

### Patient Characteristics

Overall, the 1868 study surveys received 1236 (66.17%) responses. Of the 1236 participants who responded, 1163 (94.09%) had had either telemedicine or in-person visits in the past 12 months. These participants’ mean age was 61.8 (SD 12.0) years; 4.48% (52/1161) identified as Asian, 19.72% (229/1161) as Black, 3.01% (35/1161) as Hispanic, and 72.78% (845/1161) as White. Furthermore, 69.94% (747/1068) were married, 38.73% (450/1162) had a graduate or professional degree, 70.77% (823/1163) were privately insured, and 22.96% (267/1163) were on Medicaid or Medicare. The median distance from residence to hospital was 19.9 (IQR 9.5-32.3) miles, and the median duration from cancer diagnosis to survey was 6.5 (IQR 3.6-11.0) years. By ADI quartile, patients with breast cancer living in the most socioeconomically disadvantaged neighborhoods (fourth quartile) tended to be older, Black, at a lower level of education, and on Medicaid or Medicare (Table 1).

**Table 1:** Characteristics of patients with breast cancer overall and by neighborhood socioeconomic disadvantage (n=1163).

Variable			Total (n=1163)		Area Deprivation Index^a^									*P* value^b^
					First quartile (n=381)		Second quartile (n=376)		Third quartile (n=252)		Fourth quartile (n=99)			
Age (y) at survey, mean (SD)			61.8 (12.0)		60.9 (11.5)		61.6 (11.7)		62.0 (12.9)		64.2 (12.5)		.68	
**Age (y) at survey, n (%)**														.03
	<45	107 (10.2)		33 (9.5)		37 (10.8)		26 (11.4)		7 (7.8)				
	45-54	179 (17)		64 (18.4)		59 (17.3)		38 (16.7)		12 (13.3)				
	55-64	308 (29.2)		116 (33.3)		99 (28.9)		58 (25.4)		22 (24.4)				
	≥65	460 (43.6)		135 (38.8)		147 (43)		106 (46.5)		49 (54.4)				
**Race and ethnicity, n (%)**														<.001
	Asian	52 (4.5)		26 (6.8)		14 (3.7)		6 (2.4)		3 (3)				
	Black	229 (19.7)		16 (4.2)		40 (10.7)		98 (38.9)		56 (56.6)				
	Hispanic	35 (3)		5 (1.3)		20 (5.3)		5 (2)		4 (4)				
	White	845 (72.8)		333 (87.6)		301 (80.3)		143 (56.7)		36 (36.4)				
**Highest level of education, n (%)**														<.001
	High school, GED^c^, or less	115 (9.9)		12 (3.1)		45 (12)		37 (14.7)		16 (16.2)				
	Associate’s degree or some college	259 (22.3)		52 (13.6)		86 (22.9)		70 (27.8)		44 (44.4)				
	Bachelor’s degree	338 (29.1)		127 (33.3)		102 (27.2)		69 (27.4)		20 (20.2)				
	Graduate or professional degree	450 (38.7)		190 (49.9)		142 (37.9)		76 (30.2)		19 (19.2)				
**Marital status, n (%)**														<.001
	Married	747 (69.9)		282 (80.3)		259 (73.4)		136 (59.6)		36 (40.4)				
	Single or not married	192 (18)		44 (12.5)		53 (15)		59 (25.9)		30 (33.7)				
	Divorced, separated, or widowed	129 (12.1)		25 (7.1)		41 (11.6)		33 (14.5)		23 (25.8)				
**Type of health insurance, n (%)**														<.001
	Private	823 (70.8)		302 (79.3)		276 (73.4)		162 (64.3)		49 (49.5)				
	Medicaid	50 (4.3)		5 (1.3)		8 (2.1)		17 (6.7)		15 (15.2)				
	Medicare	217 (18.7)		54 (14.2)		74 (19.7)		55 (21.8)		24 (24.2)				
	Other or unknown	73 (6.3)		20 (5.2)		18 (4.8)		18 (7.1)		11 (11.1)				
Distance from residence to hospital (miles)^d^, median (IQR)			19.9 (9.5-32.3)		20.5 (10.9-31.9)		22.5 (13.3-33.2)		16.4 (4.6-30.5)		11.9 (3.3-27.6)		<.001	
Duration (y) from cancer diagnosis to survey, median (IQR)			6.5 (3.6-11.0)		6.8 (3.7-10.9)		6.2 (3.6-10.3)		6.5 (3.6-11.5)		8.3 (4.2-11.6)		.61	
**Duration (y) from cancer diagnosis to survey, n (%)**														.63
	≤3	199 (17.1)		58 (15.2)		68 (18.1)		48 (19)		14 (14.1)				
	4-6	319 (27.4)		107 (28.1)		107 (28.5)		67 (26.6)		23 (23.2)				
	≥7	645 (55.5)		216 (56.7)		201 (53.5)		137 (54.4)		62 (62.6)				
**Charlson comorbidity index, n (%)**														.03
	0	994 (88.5)		333 (90.5)		335 (91.5)		209 (85)		77 (82.8)				
	1	62 (5.6)		19 (5.2)		11 (3)		21 (8.5)		6 (6.5)				
	≥2	67 (6.0)		16 (4.3)		20 (5.5)		16 (6.5)		10 (10.8)				
**Histologic type, n (%)**														.08
	Ductal	742 (80.2)		247 (77.9)		238 (79.9)		159 (81.1)		64 (88.9)				
	Lobular	92 (10)		38 (12)		34 (11.4)		15 (7.7)		1 (1.4)				
	Ductal and lobular	55 (6)		19 (6)		18 (6)		12 (6.1)		3 (4.2)				
	Other	36 (3.9)		13 (4.1)		8 (2.7)		10 (5.1)		4 (5.6)				
**AJCC^e^ stage group, n (%)**														.002
	0	200 (18.1)		51 (14.2)		69 (19)		51 (21.1)		21 (22.6)				
	I	515 (46.5)		189 (52.5)		160 (44.1)		104 (43)		36 (38.7)				
	II	271 (24.5)		88 (24.4)		91 (25.1)		58 (24)		24 (25.8)				
	III	112 (10.1)		31 (8.6)		42 (11.6)		24 (9.9)		10 (10.8)				
	IV	10 (0.9)		1 (0.3)		1 (0.3)		5 (2.1)		2 (2.2)				
**Molecular subtype, n (%)**														.06
	HR^f^+/HER2^g^−	571 (66.2)		208 (69.3)		180 (66.2)		120 (65.6)		35 (53.8)				
	HR+/HER+	98 (11.4)		34 (11.3)		36 (13.2)		15 (8.2)		8 (12.3)				
	HR−/HER2+	51 (5.9)		12 (4)		17 (6.2)		19 (10.4)		3 (4.6)				
	TNBC^h^	142 (16.5)		46 (15.3)		39 (14.3)		29 (15.8)		19 (29.2)				
**Tumor grade, n (%)**														.047
	1	149 (14.3)		59 (17.3)		47 (13.8)		27 (11.9)		9 (10.3)				
	2	471 (45.3)		159 (46.6)		146 (42.9)		99 (43.8)		42 (48.3)				
	3	420 (40.4)		123 (36.1)		147 (43.2)		100 (44.2)		36 (41.4)				
**Receipt of chemotherapy, n (%)**														.92
	No	572 (54.3)		190 (54.6)		182 (53.2)		125 (54.8)		48 (53.3)				
	Yes	482 (45.7)		158 (45.4)		160 (46.8)		103 (45.2)		42 (46.7)				
**Receipt of hormone therapy, n (%)**														.03
	No	341 (32.4)		100 (28.7)		113 (33)		74 (32.5)		39 (43.3)				
	Yes	713 (67.7)		248 (71.3)		229 (67)		154 (67.5)		51 (56.7)				
**Receipt of radiation therapy, n (%)**														.08
	No	394 (37.4)		140 (40.2)		125 (36.5)		83 (36.4)		26 (28.9)				
	Yes	660 (62.6)		208 (59.8)		217 (63.5)		145 (63.6)		64 (71.1)				
**Type of surgery received, n (%)**														.006
	None	13 (1.3)		5 (1.5)		3 (0.9)		2 (0.9)		3 (3.4)				
	Lumpectomy	615 (59.3)		185 (53.8)		194 (57.4)		146 (66.1)		61 (68.5)				
	Mastectomy	307 (29.6)		116 (33.7)		107 (31.7)		50 (22.6)		25 (28.1)				
	Bilateral mastectomy	102 (9.9)		38 (11.0)		34 (10.1)		23 (10.4)		0 (0)				

^a^The Area Deprivation Index (national ranking percentile) is a composite measure consisting of the domains of income, education, employment, and housing quality. It ranks neighborhoods by socioeconomic disadvantage at the national level and is scored from 1 to 100, with higher scores representing greater neighborhood socioeconomic deprivation.

^b^*P* values were calculated using Kruskal-Wallis tests.

^c^GED: General Educational Development Test.

^d^Distance from residence to hospital was calculated by taking the differences of coordinates (longitudes or latitudes) between the patient’s address and the University of Chicago Medicine Comprehensive Cancer Center’s address based on the Haversine formula.

^e^AJCC: American Joint Committee on Cancer.

^f^HR: hormone receptor.

^g^HER2: human epidermal growth factor receptor 2.

^h^TNBC: triple-negative breast cancer.

### Telemedicine Use and Association With ADI

Overall, 35.95% (416/1157) of the patients with breast cancer had a telehealth visit in the past 12 months (Table 2). By modality of telemedicine, 65% (266/409) of the clinic visits were conducted through videoconferencing only, followed by 22.7% (93/409) through telephone only and 12.2% (50/409) through both videoconferencing and telephone. The mean ADI score for telemedicine users was 37.7 (SD 24.2) compared to 39.5 (SD 24.0) for nonusers (Table 2). By ADI quartile, 38.3% (145/379) of the patients living in the least socioeconomically disadvantaged neighborhoods (first quartile) used telemedicine, followed by 37.9% (58/153), 35.1% (132/356), and 32.5% (81/249) in the fourth, second, and third quartiles, respectively. On multivariable regression analysis (model 3), higher ADI scores (per 10-unit increase) were associated with lower odds of telemedicine use (AOR 0.89, 95% CI 0.82-0.97; Table 3).

**Table 2:** Characteristics of patients with breast cancer by telehealth visit (n=1157).

Variable		Had a telehealth visit in the past 12 months		*P* value^a^	
		No (n=741)	Yes (n=416)		

**Modality of telemedicine (n=409), n (%)**					—^b^
	Telephone or audio call	—	93 (22.7)		
	Videoconference	—	266 (65)		
	Both	—	50 (12.3)		
Area Deprivation Index^c^, mean (SD)		39.5 (24.0)	37.7 (24.2)	.18	
**Area Deprivation Index, n (%)**					.13
	First quartile	234 (61.7)	145 (38.3)		
	Second quartile	224 (64.9)	132 (35.1)		
	Third quartile	168 (67.5)	81 (32.5)		
	Fourth quartile	95 (62.1)	58 (37.9)		
Age (y) at survey, mean (SD)		62.2 (11.9)	60.9 (12.2)	.09	
**Age (y) at survey, n (%)**					.04
	<45	56 (52.3)	51 (47.7)		
	45-54	121 (67.6)	58 (32.4)		
	55-64	201 (66.3)	102 (33.7)		
	≥65	299 (65.1)	160 (34.9)		
**Race and ethnicity, n (%)**					.08
	Asian	37 (71.2)	15 (28.9)		
	Black	136 (60.2)	90 (39.8)		
	Hispanic	17 (48.6)	18 (51.4)		
	White	550 (65.3)	292 (34.7)		
**Highest level of education, n (%)**					.002
	High school, GED^d^, or less	92 (80)	23 (20)		
	Associate’s degree or some college	163 (63.4)	94 (36.6)		
	Bachelor’s degree	208 (61.9)	128 (38.1)		
	Graduate or professional degree	277 (61.8)	171 (38.2)		
**Marital status, n (%)**					.70
	Married	481 (64.7)	263 (35.4)		
	Single or not married	116 (61.4)	73 (38.6)		
	Divorced, separated, or widowed	83 (64.3)	46 (35.7)		
**Type of health insurance, n (%)**					.25
	Private	515 (63)	302 (37)		
	Medicaid	28 (56)	22 (44)		
	Medicare	147 (67.7)	70 (32.3)		
	Other or unknown	51 (69.9)	22 (30.1)		
Distance (miles) from residence to hospital^e^, median (IQR)		19.9 (9.8-32.3)	20.4 (9.3-32.3)	.96	
Duration (y) from cancer diagnosis to survey, median (IQR)		6.8 (3.7-0.9)	6.3 (3.5-11.0)	.22	
**Duration (years) from cancer diagnosis to survey, n (%)**					.009
	≤3	109 (55)	89 (45)		
	4-6	217 (68)	102 (32)		
	≥7	415 (64.8)	225 (35.2)		
**Charlson comorbidity index, n (%)**					.31
	0	635 (64.3)	353 (35.7)		
	1	34 (54.8)	28 (45.2)		
	≥2	44 (65.7)	23 (34.3)		
**Histologic type, n (%)**					.27
	Ductal	459 (62.1)	280 (37.9)		
	Lobular	63 (68.5)	29 (31.5)		
	Ductal and lobular	38 (69.1)	17 (30.9)		
	Other	19 (52.8))	17 (47.2)		
**AJCC^f^ stage group, n (%)**					.26
	0	135 (68.5)	62 (31.5)		
	I	333 (64.9)	180 (35.1)		
	II	161 (59.6)	109 (40.4)		
	III	75 (61.5)	47 (38.5)		
	IV	5 (50)	5 (50)		
**Molecular subtype, n (%)**					.91
	HR^g^+/HER2^h^−	358 (62.8)	212 (37.2)		
	HR+/HER+	64 (65.3)	34 (34.7)		
	HR−/HER2+	34 (66.7)	17 (33.3)		
	TNBC^i^	87 (62.1)	53 (37.9)		
**Tumor grade, n (%)**					.10
	1	87 (59.6)	59 (40.4)		
	2	316 (67.4)	153 (32.6)		
	3	258 (61.6)	161 (38.4)		
**Receipt of chemotherapy, n (%)**					.19
	No	377 (66.4)	191 (33.6)		
	Yes	300 (62.5)	180 (37.5)		
**Receipt of hormone therapy, n (%)**					.92
	No	217 (64.4)	120 (35.6)		
	Yes	460 (64.7)	251 (35.3)		
**Receipt of radiation therapy, n (%)**					.68
	No	255 (65.4)	135 (34.6)		
	Yes	422 (64.1)	236 (35.9)		
**Type of surgery received, n (%)**					.35
	None	6 (46.2)	7 (53.8)		
	Lumpectomy	404 (66)	208 (34)		
	Mastectomy	190 (62.5)	114 (37.5)		
	Bilateral mastectomy	68 (66.7)	34 (33.3)		

^a^*P* values were calculated using 2-tailed *t* tests or Wilcoxon rank sum, Pearson chi-square, or Fisher exact tests, as appropriate.

^b^Not applicable.

^c^The Area Deprivation Index (national ranking percentile) is a composite measure consisting of the domains of income, education, employment, and housing quality. It ranks neighborhoods by socioeconomic disadvantage at the national level and is scored from 1 to 100, with higher scores representing greater neighborhood socioeconomic deprivation.

^d^GED: General Educational Development Test.

^e^Distance from residence to hospital was calculated by taking the differences of coordinates (longitudes or latitudes) between the patient’s address and the University of Chicago Medicine Comprehensive Cancer Center’s address based on the Haversine formula.

^f^AJCC: American Joint Committee on Cancer.

^g^HR: hormone receptor.

^h^HER2: human epidermal growth factor receptor 2.

^i^TNBC: triple-negative breast cancer.

In the same model (model 3), patients with breast cancer aged 45 to 54 years had lower odds of having a telehealth visit than those aged <45 years (AOR 0.49, 95% CI 0.27-0.91). Patients aged 55 to 64 years (AOR 0.63, 95% CI 0.36-1.12) or ≥65 years (AOR 0.63, 95% CI 0.34-1.18) also had a lower likelihood, but these differences were not statistically significant. Black (AOR 2.38, 95% CI 1.41-4.00) or Hispanic (AOR 2.65, 95% CI 1.07-6.58) patients had greater odds of telemedicine use than White patients. Compared to patients with high school or less education, those with an associate’s (AOR 2.67, 95% CI 1.33-5.35), bachelor’s (AOR 2.75, 95% CI 1.38-5.48), or graduate (AOR 2.57, 95% CI 1.31-5.04) degree had higher odds of telemedicine use in the past 12 months. Longer distance from residence to hospital (per 10-mile increase) was associated with greater odds of use of telemedicine, although this was not statistically significant (AOR 1.02, 95% CI 0.96-1.09; Table 3). Clinicopathologic and treatment factors were not significantly associated with telemedicine use (Table S1 in Multimedia Appendix 2). In subgroup analyses, ADI scores were not significantly different between videoconference and telephone visits (AOR 0.88, 95% CI 0.73-1.07). We also observed that patients with a graduate or professional degree had greater odds of using videoconference visits (AOR 5.78, 95% CI 1.03-32.55), and patients on Medicare had lower odds of videoconference visit use than privately insured patients (AOR 0.26, 95% CI 0.07-0.91; Table S2 in Multimedia Appendix 2).

**Table 3:** Association between neighborhood socioeconomic disadvantage and telemedicine use in patients with breast cancer.

Variable		Model 1, adjusted odds ratio^a^ (95% CI)	*P* value	Model 2, adjusted odds ratio^b^ (95% CI)	*P* value	Model 3, adjusted odds ratio^c^ (95% CI)	*P* value
Area Deprivation Index^d^ (continuous)^e^		0.93 (0.87-0.99)	.03	0.89 (0.82-0.96)	.005	0.89 (0.82-0.97)	.004
Distance from residence to hospital^e^		1.04 (0.99-1.10)	.13	1.03 (0.97-1.09)	.40	1.02 (0.96-1.09)	.48
**Age (y) at survey**							
	<45	1.0 (reference)		1.0 (reference)		1.0 (reference)	
	45-54	0.55 (0.33-0.94)	.03	0.53 (0.29-0.97)	.04	0.49 (0.27-0.91)	.02
	55-64	0.57 (0.35-0.93)	.02	0.64 (0.37-1.11)	.11	0.63 (0.36-1.12)	.11
	≥65	0.65 (0.39-1.09)	.10	0.62 (0.34-1.13)	.12	0.63 (0.34-1.18)	.16
**Race and ethnicity**							
	Asian	0.55 (0.26-1.17)	.12	0.50 (0.20-1.22)	.13	0.50 (0.20-1.23)	.16
	Black	1.86 (1.21-2.86)	.005	2.50 (1.48-4.20)	.001	2.38 (1.41-4.00)	.001
	Hispanic	2.12 (1.02-4.41)	.04	2.85 (1.17-6.91)	.02	2.65 (1.07-6.58)	.03
	White	1.0 (reference)		1.0 (reference)		1.0 (reference)	
**Highest level of education**							
	High school, GED^f^, or less	1.0 (reference)		1.0 (reference)		1.0 (reference)	
	Associate’s degree or some college	2.66 (1.47-4.81)	.001	2.76 (1.40-5.44)	.003	2.67 (1.33-5.35)	.006
	Bachelor’s degree	2.43 (1.35-4.38)	.003	2.61 (1.33-5.10)	.005	2.75 (1.38-5.48)	.004
	Graduate or professional degree	2.46 (1.39-4.38)	.002	2.55 (1.32-4.93)	.005	2.57 (1.31-5.04)	.006
**Duration (y) from cancer diagnosis to survey**							
	≤3	1.0 (reference)		1.0 (reference)		1.0 (reference)	
	4-6	0.63 (0.41-0.96)	.03	0.67 (0.42-1.08)	.12	0.75 (0.46-1.21)	.24
	≥7	0.67 (0.45-1.01)	.05	0.60 (0.38-0.96)	.04	0.65 (0.40-1.05)	.09

^a^Additionally adjusted for marital status, health insurance, and Charlson comorbidity index.

^b^Additionally adjusted for marital status, health insurance, Charlson comorbidity index, histologic type, stage, molecular subtype, and tumor grade.

^c^Additionally adjusted for marital status; type of health insurance; Charlson comorbidity index; histologic type; stage; molecular subtype; tumor grade; receipt of chemotherapy, hormone therapy, or radiotherapy; and type of surgery.

^d^The Area Deprivation Index (national ranking percentile) is a composite measure consisting of the domains of income, education, employment, and housing quality. It ranks neighborhoods by socioeconomic disadvantage at the national level and is scored from 1 to 100, with higher scores representing greater neighborhood socioeconomic deprivation.

^e^Odds ratios were per 10-unit increase.

^f^GED: General Educational Development Test.

### Comparisons of Provisions of Oncology Services Between Telehealth and In-Person Visits

**Figure 1:**
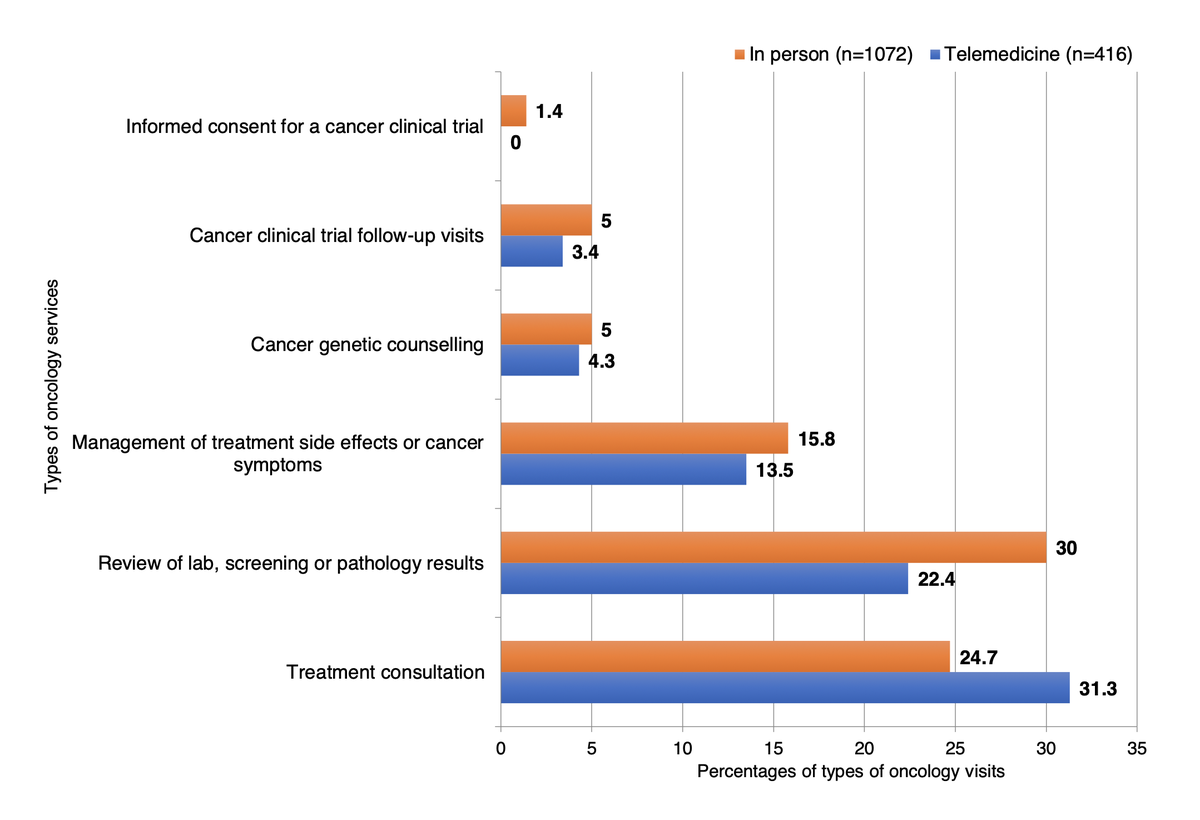
Percentages of oncology services by type of visit among patients with breast cancer.

Figure 1 displays the breakdown of oncology services by visit type for the patients with breast cancer. Overall, 31.3% (130/416) of the patients used telemedicine for the purpose of treatment consultation; 22.4% (93/416) for reviewing laboratory, screening, and pathology test results; 13.5% (56/416) for managing cancer symptoms and treatment side effects; 4.3% (18/416) for cancer genetic counseling; and 3.4% (14/416) for cancer clinical trial follow-ups. Among patients who had in-person visits, reviewing laboratory, screening, and pathology test results was reported the most (322/1072, 30.04%), followed by treatment consultation (265/1072, 24.72%), management of cancer symptoms and treatment side effects (169/1072, 15.76%), genetic counseling (54/1072, 5.04%), and cancer clinical trial follow-ups (54/1072, 5.04%). After analyzing match-paired data (Table 4), we observed significant differences between telemedicine and in-person visits in the provision of management of cancer symptoms and treatment side effects; review of laboratory, screening, and pathology test results; and cancer clinical trial follow-ups. However, there were no significant differences in offering treatment consultation (45/405, 11.1% vs 55/405, 13.6%; *P*=.32) or cancer genetic counseling (11/405, 2.7% vs 19/405, 4.7%; *P*=.14) between telehealth and in-person visits (Table 4).

**Figure 2:**
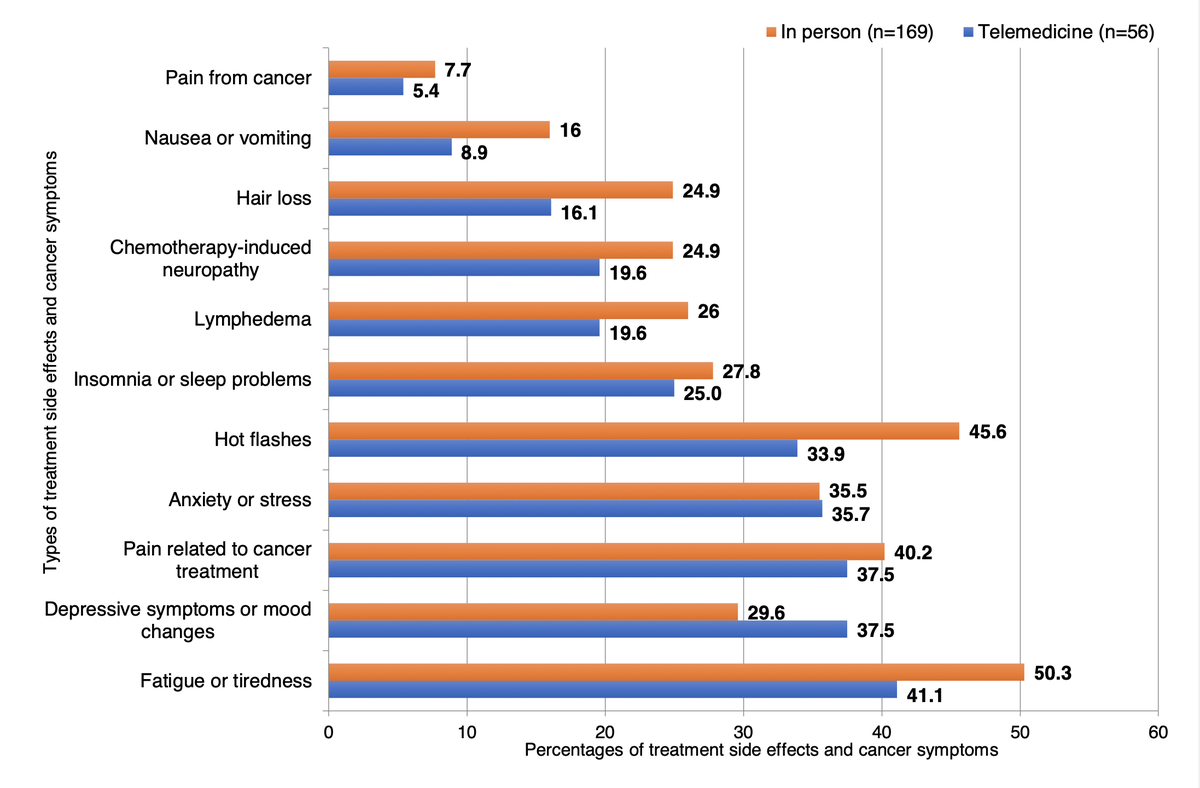
Percentages of cancer symptoms and treatment side effects discussed during telemedicine or in-person visits reported by patients with breast cancer.

Among the patients with breast cancer who reported management of cancer symptoms and treatment side effects (Figure 2), those with in-person visits had greater proportions of instances of discussions of fatigue (85/169, 50.3% vs 23/56, 41%), hot flashes (77/169, 45.6% vs 19/56, 34%), lymphedema (44/169, 26% vs 11/56, 20%), chemotherapy-induced neuropathy (42/169, 24.9% vs 11/56, 20%), or nausea and vomiting (27/169, 16% vs 5/56, 9%) than patients with telehealth visits, whereas a higher proportion of patients had discussed depressive symptoms through telemedicine than in-person visits (21/56, 38% vs 50/169, 29.6%). By modality of telemedicine, a higher percentage of patients used both telephone and video visits for treatment consultation than video visit or telephone visit alone (Table S3 in Multimedia Appendix 2). In addition, there were no significant differences in the distributions of various methods of managing cancer symptoms and treatment side effects across the 3 telemedicine modalities (Table S4 in Multimedia Appendix 2).

**Table 4:** Analysis of match-paired data on oncology services by type of visit among patients with breast cancer (n=405).

Type of visit and variable				Telemedicine visits, n (%)					*P* value^a^
				No		Yes			

**In-person visits, n (%)**									
	**Treatment consultation**								.32
		No	222 (54.8)		45 (11.1)				
		Yes	55 (13.6)		83 (20.5)				
	**Management of treatment side effects or cancer symptoms**								<.001
		No	295 (72.8)		15 (3.7)				
		Yes	54 (13.3)		41 (10.1)				
	**Review of laboratory, screening, and pathology test results**								<.001
		No	224 (55.3)		26 (6.4)				
		Yes	90 (22.2)		65 (16)				
	**Cancer genetic counseling**								.14
		No	368 (90.9)		11 (2.7)				
		Yes	19 (4.7)		7 (1.7)				
	**Cancer clinical trial follow-up visits**								.046
		No	379 (93.6)		4 (1)				
		Yes	12 (3)		10 (2.5)				

^a^*P* values were calculated using the McNemar test.

### Telemedicine Use Challenges or Concerns and Satisfaction

Compared with the patients with breast cancer who did not use telemedicine, those who did reported a higher percentage of technology difficulty or lack of comfort with technology (51/416, 12.3% vs 21/741, 2.8%; *P*<.001), compromised patient-provider communication (28/416, 6.7% vs 16/741, 2.2%; *P*<.001), and compromised patient-provider relationship (35/416, 8.4% vs 12/741, 1.6%; *P*<.001; Table 5). Furthermore, of the telemedicine users, 93.7% (132/141) and 95.5% (301/315) reported being somewhat to extremely satisfied with their telephone visit and videoconference visit experiences, respectively, and 61.8% (254/411) were likely or very likely to continue using telemedicine (Table S5 in Multimedia Appendix 2). In addition, no significant differential distributions of satisfaction with telehealth visits across all oncology services were observed, stratified by modality of telemedicine (Tables 6 and 7) or overall (Table S6 in Multimedia Appendix 2).

**Table 5:** Percentages of challenges or concerns regarding telemedicine use reported by patients with breast cancer (n=1157).

Variables			Overall (n=1157), n (%)		Had a telehealth visit in the past 12 months					*P* value^a^
					No (n=741), n (%)		Yes (n=416), n (%)			

**Technology difficulty or lack of comfort with technology**										<.001
	No	1085 (93.8)		720 (97.2)		365 (87.7)				
	Yes	72 (6.2)		21 (2.8)		51 (12.3)				
**Lack of an electronic device (eg, desktop computer, laptop computer, smartphone, or iPad)**										.20
	No	1151 (99.5)		739 (99.7)		412 (99)				
	Yes	6 (0.5)		2 (0.3)		4 (1)				
**Lack of high-speed internet or slow internet connection at home**										.06
	No	1133 (97.9)		730 (98.5)		403 (96.9)				
	Yes	24 (2.1)		11 (1.5)		13 (3.1)				
**Compromised patient-provider communication**										<.001
	No	1113 (96.2)		725 (97.8)		388 (93.3)				
	Yes	44 (3.8)		16 (2.2)		28 (6.7)				
**Compromised patient-provider relationship**										<.001
	No	1110 (95.9)		729 (98.4)		381 (91.6)				
	Yes	47 (4.1)		12 (1.6)		35 (8.4)				
**Not being offered at the clinic or by a provider**										.80
	No	1140 (98.5)		729 (98.4)		411 (98.8)				
	Yes	17 (1.5)		12 (1.6)		5 (1.2)				
**Cost**										.30
	No	1148 (99.2)		737 (99.5)		411 (98.8)				
	Yes	9 (0.8)		4 (0.5)		5 (1.2)				
**Not being covered by health insurance**										.22
	No	1146 (99)		736 (99.3)		410 (98.6)				
	Yes	11 (1)		5 (0.7)		6 (1.4)				

^a^*P* values were calculated using Pearson chi-square or Fisher exact tests.

**Table 6:** Percentages of satisfaction with telephone visit by oncology service among patients with breast cancer (n=141).

Were your telemedicine visits related to the following oncology services?			How satisfied were you with your telephone visit with physicians or other health care providers?											*P* value^a^	
			Not at all (n=2), n (%)		A little (n=7), n (%)		Somewhat (n=28), n (%)		Very (n=62), n (%)		Extremely (n=42), n (%)				

**Treatment consultation**															.70
	No	1 (1)		5 (5)		20 (19.8)		44 (43.6)		31 (30.7)					
	Yes	1 (2)		2 (5)		8 (20)		18 (45)		11 (27.5)					
**Review of laboratory, screening, and pathology test results**															.92
	No	1 (1.0)		3 (3.0)		25 (24.8)		41 (40.6)		31 (30.7)					
	Yes	1 (2.5)		4 (10.0)		3 (7.5)		21 (52.5)		11 (27.5)					
**Management of treatment side effects or cancer symptoms**															.22
	No	1 (0.8)		7 (5.7)		24 (19.5)		51 (41.5)		40 (32.5)					
	Yes	1 (5.6)		0 (0)		4 (22.2)		11 (61.1)		2 (11.1)					
**Cancer genetic counseling**															.58
	No	2 (1.5)		7 (5.3)		26 (19.6)		57 (42.9)		41 (30.8)					
	Yes	0 (0)		0 (0)		2 (25)		5 (62.5)		1 (12.5)					
**Cancer clinical trial follow-up visits**															.95
	No	2 (1.4)		7 (5.0)		28 (20.0)		61 (43.6)		42 (300)					
	Yes	0 (0)		0 (0)		0 (0)		1 (100)		0 (0)					

^a^*P* values were calculated using Wilcoxon rank sum tests.

**Table 7:** Percentages of satisfaction with videoconference visit by oncology service among patients with breast cancer (n=315).

Were your telemedicine visits related to the following oncology services?		How satisfied were you with your videoconference visit with physicians or other health care providers?						*P* value^a^	
		Not at all (n=8), n (%)	A little (n=6), n (%)	Somewhat (n=56), n (%)	Very (n=157), n (%)	Extremely (n=88), n (%)			

**Treatment consultation**									.78
	No	4 (2)	5 (2.5)	36 (17.7)	104 (51)	55 (27)			
	Yes	4 (3.6)	1 (0.9)	20 (18)	53 (47.8)	33 (29.7)			
**Review of laboratory, screening, and pathology test results**									.77
	No	6 (2.4)	4 (1.6)	47 (19.1)	117 (47.6)	72 (29.3)			
	Yes	2 (2.9)	2 (2.9)	9 (13)	40 (58)	16 (23.2)			
**Management of treatment side effects or cancer symptoms**									.20
	No	8 (3)	6 (2.2)	50 (18.5)	132 (48.9)	74 (27.4)			
	Yes	0 (0)	0 (0)	6 (13.3)	25 (55.6)	14 (31.1)			
**Cancer genetic counseling**									.98
	No	7 (2.3)	6 (2)	54 (17.9)	150 (49.8)	84 (27.9)			
	Yes	1 (7.1)	0 (0)	2 (14.3)	7 (50)	4 (28.6)			
**Cancer clinical trial follow-up visits**									.48
	No	8 (2.7)	6 (2)	54 (17.9)	146 (48.5)	87 (28.9)			
	Yes	0 (0)	0 (0)	2 (14.3)	11 (78.6)	1 (7.1)			

^a^*P* values were calculated using Wilcoxon rank sum tests.

## Discussion

### Principal Findings

This study built on previous work investigating the relationship between neighborhood socioeconomic disadvantage and telemedicine use among patients with breast cancer and comparing provisions of oncology services between telehealth and in-person office visits. Telemedicine use has expanded dramatically since the COVID-19 pandemic began. However, not all patients with breast cancer benefited from the expansion; as our study uncovered, patients with higher ADI scores (ie, lower neighborhood-level SES) were less likely to have used telemedicine in the past 12 months. Although patients cited technology difficulty or lack of comfort with technology as well as compromised patient-provider communication and compromised patient-provider relationship as common challenges or concerns, they expressed high degrees of satisfaction with telehealth experiences and willingness to continue using telehealth care and services in the future. In addition, both telemedicine and in-person visits were equally likely to deliver treatment consultation and cancer genetic counseling, while services that were more likely to be offered in person were management of cancer symptoms and treatment side effects; review of laboratory, screening, and pathology test results; and cancer clinical trial follow-ups.

One key study finding is that patients with breast cancer living in greater socioeconomically disadvantaged neighborhoods were less likely to use telemedicine for care and services, consistent with previous studies of patients in primary care, adult outpatient, and hematology or oncology settings [8,24-28]. A descriptive study suggested no correlation between ADI scores and interest in telehealth visits among 64 patients with head and neck cancers (interested: median ADI score=4.0 vs not interested: median ADI score=5.0; *P*=.79) [27], but the small sample size limited the reliability of these findings. Lee et al [28] analyzed a cohort of 341,089 patients with hematologic malignancies and patients with cancer, demonstrating a significant difference in the distribution of telemedicine use between patients living in the most socioeconomically disadvantaged neighborhoods and those living in the least socioeconomically disadvantaged neighborhoods (46.1% vs 54%) [28]. However, the proportion of patients with breast cancer as well as adjusted estimates are unknown due to the descriptive nature of this analysis. In another study, Hassan et al [8] observed a 3% decrease in the number of telemedicine visits per 10-unit increase in the ADI score among patients with cancer who were financially distressed, whereas an 11% decrease in telemedicine use was observed in our cohort of patients with breast cancer. Our estimated effect is larger possibly due to our large sample size and the heterogeneous group of patients with breast cancer. Our findings indicate that neighborhood-level SES may have a bigger impact on the use of telemedicine among patients with breast cancer. Neighborhood socioeconomic disadvantage is associated with lower access to telehealth care and services. Strategies to enhance the accessibility of telehealth are needed to reduce neighborhood-level socioeconomic disparities in telemedicine use among patients with breast cancer, particularly among patients living in socioeconomically deprived neighborhoods and regions.

Consistent with prior research in patients with breast, gynecologic, or other cancers [3,4,11], we found that >1 in 3 patients with breast cancer had had a telehealth visit in the past 12 months. With respect to race and ethnicity, Black or Hispanic patients were more than twice as likely as their White counterparts to have used telemedicine. In previous studies, Campos-Castillo and Anthony [44] observed that Black or Latinx American adults were more likely to have telehealth visits, and Reed et al [45] documented a higher likelihood of patients using both telephone and video visits for primary care. However, a study at an outpatient oncology care center revealed that Asian or Hispanic patients were less likely than White patients to have used telemedicine, while no significant difference existed between Black and White patients [11]. These inconsistent results are possibly due to sampling variability and the heterogeneous characteristics of patient populations in oncology and other clinical settings. Nevertheless, our finding indicates that patients belonging to racial and ethnic minority groups with breast cancer may have unique and greater needs for telehealth. Similarly, compared to patients with high school or less education, those with an associate’s, bachelor’s, or graduate degree had >2-fold greater odds of telemedicine use. Older age groups were associated with a lower likelihood of telemedicine use. These findings are well documented in the literature and highlight the influence of individual-level SES on the use of telemedicine. Telemedicine may have the potential to improve telehealth care and service use among patients belonging to racial and ethnic minority groups with breast cancer. Oncology programs should also ensure that patients who are older and those with lower levels of education have equal access to telehealth care and services.

Another notable finding is that patient-reported treatment consultations and cancer genetic counseling services occur with an equal frequency when comparing telemedicine and in-person office visits. Our study supports existing literature on the feasibility and acceptability of teleoncology consultations and telehealth-based cancer genetic counseling among providers and their patients with cancer [7,31-42]. However, only a few prior studies compared these oncology services between telemedicine and in-person visits, and most samples were small. A univariate analysis of 509 patients with cancer from the University of Texas MD Anderson Cancer Center reported a similar distribution of patients seeking integrative oncology consultation between telehealth and in-person settings [37]. McDonald et al [46] illustrated no significant differences in providing cancer genetic services (in-person vs telegenetics) by residential area or perceived cancer risk. We also observed that patients were particularly more likely to join treatment consultations through videoconference than telephone. However, we found significant variations between telehealth and in-person office visits in provisions of management of cancer symptoms and treatment side effects; review of laboratory, screening, and pathology test results; and cancer clinical trial follow-ups. In the subgroup of patients with breast cancer who reported management of cancer symptoms and treatment side effects, more than one-third of the patients discussed depressive symptoms or mood change (23/56, 41%) or anxiety or stress (20/56, 36%) during telehealth visits with their providers. This finding indicates that telemedicine may be in greater need among these patients. Taken together, our data demonstrate the variations in provisions of oncology services between telemedicine and in-person visits among patients with breast cancer. Cancer programs and providers may consider tailoring the delivery of care and services according to patients’ care needs and preferences. Future research will be necessary to explore reasons or factors that explain these variations and whether there are unmet telehealth needs in patients with breast cancer experiencing certain mental health symptoms.

With regard to challenges or concerns related to telemedicine use, we found that significantly higher proportions of the patients with breast cancer who had telehealth visits cited technology difficulty or lack of comfort with technology, compromised patient-provider communication, or compromised patient-provider relationship. Possibly, the telemedicine nonusers in our study did not have firsthand experience of technology difficulty. Our finding is aligned with previous research [27] but not with 2 cross-sectional studies [9,38] that reported similar patient-provider communication experiences when comparing telehealth and in-person visits. Analyses are further needed to determine the correlations between telemedicine use and these challenges or concerns among patients with breast cancer. Despite these challenges or concerns, patients reported a remarkably high level of satisfaction with their telemedicine use experiences (390/407, 95.8%), and 61.8% (254/411) expressed the willingness to continue telemedicine use, congruent with prior studies in patients with cancer [6,7,10,33,34]. However, prior research evaluated only overall satisfaction, whereas we found a similar level of satisfaction by type of oncology service or across various cancer symptoms and treatment side effects discussed during telehealth visits, irrespective of the modality of telemedicine. These findings offer a practical implication for telehealth implementation and care and service delivery, but future research in telemedicine program evaluation is necessary.

### Limitations

Several limitations of this study should be considered. First, the survey data were per self-report, which is prone to recall error or social desirability. However, this bias is likely very minimal because our research staff had limited to no interaction with the participants that would influence the survey responses. Second, the study sample included only patients with breast cancer who were willing to respond to our survey. The proportions of self-reported visits for oncology services and perceived challenges or concerns related to telemedicine use have probably been underestimated. Our estimated effect of neighborhood socioeconomic disadvantage on telehealth use in patients with breast cancer may also be underestimated. Third, this study evaluated broader access to telehealth care and services, including oncology; therefore, the results may not be specific to breast oncology. However, it is important to point out that, regardless of treatment status and duration since diagnosis, patients have other care and service needs across the cancer care continuum and different clinical settings. Fourth, distance from residence to hospital was not associated with telemedicine use. It was calculated based on the Haversine formula, which did not account for travel time, traffic patterns, lack of transportation, road conditions, weather, and other environmental factors. In addition, we were not able to assess other unmeasured potential confounding factors, such as the availability and density of telehealth clinics or cancer programs in the geographic area and local technology or digital infrastructure, that could affect the associations or variability we observed in this analysis. Thus, this warrants future research. Finally, participants in the ChiMEC study may not be representative of all patients with breast cancer nationally, which limits the generalizability of our findings.

### Conclusions

In conclusion, our findings from ChiMEC patients with breast cancer offer insights into the impact of neighborhood socioeconomic disadvantage on telemedicine use and comparing provisions of oncology services between telehealth and in-person office visits, underscoring the importance of identifying neighborhood-level socioeconomic disparities and patients’ unmet needs for telemedicine. Oncology programs should address these disparities and needs to improve care delivery and achieve telehealth equity for their patient populations. Meanwhile, as cancer centers and research organizations further embrace telemedicine and other digital platforms, it is essential to tackle patients’ perceived challenges or concerns and consider allocating these platforms to particular care and services, such as genetic counseling, treatment consultation, and management of depressive symptoms and anxiety, to provide high-quality telehealth care and services to patients with breast cancer.

## Appendix

Multimedia Appendix 1 Survey questions regarding telehealth and in-person office visits.

Multimedia Appendix 2 Associations between neighborhood socioeconomics disadvantage and telemedicine use (video conference versus telephone); comparing provisions of oncology services by modality of telemedicine; and patient satisfaction with telemedicine.

## Abbreviations

ADI: Area Deprivation Index
AOR: adjusted odds ratio
ChiMEC: Chicago Multiethnic Epidemiologic Breast Cancer Cohort
SES: socioeconomic status

## Data Availability

The data sets generated and analyzed during this study are not publicly available due to patient confidentiality and privacy concerns but are available from the corresponding author on reasonable request.

## References

[1] Fact sheet: telehealth. American Hospital Association.

[2] Koonin LM, Hoots B, Tsang CA, Leroy Z, Farris K, Jolly B, Antall P, McCabe B, Zelis CB, Tong I, Harris AM (2020). Trends in the use of telehealth during the emergence of the COVID-19 pandemic - United States, January-March 2020. MMWR Morb Mortal Wkly Rep.

[3] Zimmerman BS, Seidman D, Berger N, Cascetta KP, Nezolosky M, Trlica K, Ryncarz A, Keeton C, Moshier E, Tiersten A (2020). Patient perception of telehealth services for breast and gynecologic oncology care during the COVID-19 pandemic: a single center survey-based study. J Breast Cancer.

[4] Lucas JW, Villarroel MA (2022). Telemedicine use among adults : United States, 2021. Centers for Disease Control and Prevention.

[5] Sonagli M, Cagnacci Neto R, Leite FP, Makdissi FB (2021). The use of telemedicine to maintain breast cancer follow-up and surveillance during the COVID-19 pandemic. J Surg Oncol.

[6] Johnson BA, Lindgren BR, Blaes AH, Parsons HM, LaRocca CJ, Farah R, Hui JY (2021). The new normal? patient satisfaction and usability of telemedicine in breast cancer care. Ann Surg Oncol.

[7] Bizot A, Karimi M, Rassy E, Heudel PE, Levy C, Vanlemmens L, Uzan C, Deluche E, Genet D, Saghatchian M, Giacchetti S, Grenier J, Patsouris A, Dieras V, Pierga JY, Petit T, Ladoire S, Jacot W, Benderra MA, De Jesus A, Delaloge S, Lambertini M, Pistilli B (2021). Multicenter evaluation of breast cancer patients' satisfaction and experience with oncology telemedicine visits during the COVID-19 pandemic. Br J Cancer.

[8] Hassan AM, Chu CK, Liu J, Angove R, Rocque G, Gallagher KD, Momoh AO, Caston NE, Williams CP, Wheeler S, Butler CE, Offodile AC (2022). Determinants of telemedicine adoption among financially distressed patients with cancer during the COVID-19 pandemic: insights from a nationwide study. Support Care Cancer.

[9] Ludwigson A, Huynh V, Myers S, Hampanda K, Christian N, Ahrendt G, Romandetti K, Tevis S (2022). Patient perceptions of changes in breast cancer care and well-being during COVID-19: a mixed methods study. Ann Surg Oncol.

[10] Freeman JQ, Khwaja A, Zhao F, Nanda R, Olopade OI, Huo D (2024). Racial/ethnic disparities in telemedicine utilization and satisfaction among breast cancer patients during the COVID-19 pandemic: a mixed-methods analysis. Telemed J E Health.

[11] Qian AS, Schiaffino MK, Nalawade V, Aziz L, Pacheco FV, Nguyen B, Vu P, Patel SP, Martinez ME, Murphy JD (2022). Disparities in telemedicine during COVID-19. Cancer Med.

[12] Kircher SM, Mulcahy M, Kalyan A, Weldon CB, Trosman JR, Benson AB (2020). Telemedicine in oncology and reimbursement policy during COVID-19 and beyond. J Natl Compr Canc Netw.

[13] (2020). Medicare telemedicine health care provider fact sheet. U.S. Centers for Medicare & Medicaid Services.

[14] Zon RT, Kennedy EB, Adelson K, Blau S, Dickson N, Gill D, Laferriere N, Lopez AM, Mulvey TM, Patt D, Pickard TA, Purdom T, Royce TJ, Sumrall AL, Page RD (2021). Telehealth in oncology: ASCO standards and practice recommendations. JCO Oncol Pract.

[15] Social determinants of health. U.S. Department of Health and Human Services.

[16] Kolak M, Bhatt J, Park YH, Padrón NA, Molefe A (2020). Quantification of neighborhood-level social determinants of health in the continental United States. JAMA Netw Open.

[17] Rosenzweig MQ, Althouse AD, Sabik L, Arnold R, Chu E, Smith TJ, Smith K, White D, Schenker Y (2021). The association between area deprivation index and patient-reported outcomes in patients with advanced cancer. Health Equity.

[18] Fairfield KM, Black AW, Ziller EC, Murray K, Lucas FL, Waterston LB, Korsen N, Ineza D, Han PK (2020). Area deprivation index and rurality in relation to lung cancer prevalence and mortality in a rural state. JNCI Cancer Spectr.

[19] Kurani SS, McCoy RG, Lampman MA, Doubeni CA, Finney Rutten LJ, Inselman JW, Giblon RE, Bunkers KS, Stroebel RJ, Rushlow D, Chawla SS, Shah ND (2020). Association of neighborhood measures of social determinants of health with breast, cervical, and colorectal cancer screening rates in the US Midwest. JAMA Netw Open.

[20] Major JM, Norman Oliver M, Doubeni CA, Hollenbeck AR, Graubard BI, Sinha R (2012). Socioeconomic status, healthcare density, and risk of prostate cancer among African American and Caucasian men in a large prospective study. Cancer Causes Control.

[21] Unger JM, Moseley AB, Cheung CK, Osarogiagbon RU, Symington B, Ramsey SD, Hershman DL (2021). Persistent disparity: socioeconomic deprivation and cancer outcomes in patients treated in clinical trials. J Clin Oncol.

[22] Hassan AM, Nguyen HT, Corkum JP, Liu J, Kapur SK, Chu CK, Tamirisa N, Offodile AC (2023). Area deprivation index is associated with variation in quality of life and psychosocial well-being following breast cancer surgery. Ann Surg Oncol.

[23] Arcaya MC, Tucker-Seeley RD, Kim R, Schnake-Mahl A, So M, Subramanian SV (2016). Research on neighborhood effects on health in the United States: a systematic review of study characteristics. Soc Sci Med.

[24] Ostovari M, Zhang Z, Patel V, Jurkovitz C (2023). Telemedicine and health disparities: association between the area deprivation index and primary care telemedicine utilization during the COVID-19 pandemic. J Clin Transl Sci.

[25] Brown SH, Griffith ML, Kripalani S, Horst SN (2022). Association of health literacy and area deprivation with initiation and completion of telehealth visits in adult medicine clinics across a large health care system. JAMA Netw Open.

[26] Hsiao V, Chandereng T, Lankton RL, Huebner JA, Baltus JJ, Flood GE, Dean SM, Tevaarwerk AJ, Schneider DF (2021). Disparities in telemedicine access: a cross-sectional study of a newly established infrastructure during the COVID-19 pandemic. Appl Clin Inform.

[27] Fassas S, Cummings E, Sykes KJ, Bur AM, Shnayder Y, Kakarala K (2021). Telemedicine for head and neck cancer surveillance in the COVID-19 era: promise and pitfalls. Head Neck.

[28] Lee MJ, Lyon L, Conell CA, Sun H, Anderson B, Neeman E, Kumar D, Kotak D, Shiraz A, Reed M, Liu R (2023). Trends and disparities in oncology telehealth after the initial pandemic era. J Clin Oncol.

[29] Kind AJ, Buckingham WR (2018). Making neighborhood-disadvantage metrics accessible — the neighborhood atlas. N Engl J Med.

[30] 2015 Area deprivation index v2.0. University of Wisconsin School of Medicine Public Health.

[31] Hsiehchen D, Muquith M, Haque W, Espinoza M, Yopp A, Beg MS (2021). Clinical efficiency and safety outcomes of virtual care for oncology patients during the COVID-19 pandemic. JCO Oncol Pract.

[32] Chávarri-Guerra Y, Ramos-López WA, Covarrubias-Gómez A, Sánchez-Román S, Quiroz-Friedman P, Alcocer-Castillejos N, Del Pilar Milke-García M, Carrillo-Soto M, Morales-Alfaro A, Medina-Palma M, Aguilar-Velazco JC, Morales-Barba K, Razcon-Echegaray A, Maldonado J, Soto-Perez-de-Celis E (2021). Providing supportive and palliative care using telemedicine for patients with advanced cancer during the COVID-19 pandemic in Mexico. Oncologist.

[33] Nandra K, Koenig G, DelMastro A, Mishler EA, Hollander JE, Yeo CJ (2019). Telehealth provides a comprehensive approach to the surgical patient. Am J Surg.

[34] Chang PJ, Jay GM, Kalpakjian C, Andrews C, Smith S (2021). Patient and provider-reported satisfaction of cancer rehabilitation telemedicine visits during the COVID-19 pandemic. PM R.

[35] Kummerow Broman K, Vella MA, Tarpley JL, Dittus RS, Roumie CL (2016). Identification of postoperative care amenable to telehealth. Surgery.

[36] Hwa K, Wren SM (2013). Telehealth follow-up in lieu of postoperative clinic visit for ambulatory surgery: results of a pilot program. JAMA Surg.

[37] Narayanan S, Lopez G, Powers-James C, Fellman BM, Chunduru A, Li Y, Bruera E, Cohen L (2021). Integrative oncology consultations delivered via telehealth in 2020 and in-person in 2019: paradigm shift during the COVID-19 world pandemic. Integr Cancer Ther.

[38] Street RL Jr, Treiman K, Kranzler EC, Moultrie R, Arena L, Mack N, Garcia R (2022). Oncology patients' communication experiences during COVID-19: comparing telehealth consultations to in-person visits. Support Care Cancer.

[39] Brown J, Athens A, Tait DL, Crane EK, Higgins RV, Naumann RW, Gusic LH, Amacker-North L (2018). A comprehensive program enabling effective delivery of regional genetic counseling. Int J Gynecol Cancer.

[40] Mette LA, Saldívar AM, Poullard NE, Torres IC, Seth SG, Pollock BH, Tomlinson GE (2016). Reaching high-risk underserved individuals for cancer genetic counseling by video-teleconferencing. J Community Support Oncol.

[41] Pruthi S, Stange KJ, Malagrino GD Jr, Chawla KS, LaRusso NF, Kaur JS (2013). Successful implementation of a telemedicine-based counseling program for high-risk patients with breast cancer. Mayo Clin Proc.

[42] Solomons NM, Lamb AE, Lucas FL, McDonald EF, Miesfeldt S (2018). Examination of the patient-focused impact of cancer telegenetics among a rural population: comparison with traditional in-person services. Telemed J E Health.

[43] Zhao F, Copley B, Niu Q, Liu F, Johnson JA, Sutton T, Khramtsova G, Sveen E, Yoshimatsu TF, Zheng Y, Ibraheem A, Jaskowiak N, Nanda R, Fleming GF, Olopade OI, Huo D (2021). Racial disparities in survival outcomes among breast cancer patients by molecular subtypes. Breast Cancer Res Treat.

[44] Campos-Castillo C, Anthony D (2021). Racial and ethnic differences in self-reported telehealth use during the COVID-19 pandemic: a secondary analysis of a US survey of internet users from late March. J Am Med Inform Assoc.

[45] Reed ME, Huang J, Graetz I, Lee C, Muelly E, Kennedy C, Kim E (2020). Patient characteristics associated with choosing a telemedicine visit vs office visit with the same primary care clinicians. JAMA Netw Open.

[46] McDonald E, Lamb A, Grillo B, Lucas L, Miesfeldt S (2014). Acceptability of telemedicine and other cancer genetic counseling models of service delivery in geographically remote settings. J Genet Couns.

