# Supplementary material for: Assessing the Relationship Between Neighborhood Socioeconomic Disadvantage and Telemedicine Use Among Patients With Breast Cancer and Examining Differential Provisions of Oncology Services Between Telehealth and In-Person Visits: Quantitative Study
Source: JMIR Cancer. 2024 Jul 18;10:e55438. doi: 10.2196/55438 (PMC11294759; doi:10.2196/55438)
Supplement: Multimedia Appendix 1 [file cancer_v10i1e55438_app1.docx]

**Supplemental Material**

**Survey Questions** – Section: Telemedicine and In-Person Visits

***Survey Questions***

***Section: Telemedicine and In-Person Visits***

1. In the past 12 months, have you scheduled in-person appointments and visits with a doctor or other health providers?

□ Yes □ No

2. [If “yes” to question #1] Did you have difficulty with making an appointment?

□ Yes □ No

3. [If “yes” to question #1] Were your in-person visits related to_________? Select all that apply.

□ Treatment consultation

□ Management of treatment side effects or cancer symptoms

□ Review of laboratory, screening, and/or pathology results

□ Cancer genetic counseling

□ Informed consent for a cancer clinical trial

□ Cancer clinical trial follow-up visits

□ Other, please specify: __________

4. In the past 12 months, have you scheduled telemedicine (a.k.a. virtual) appointments and visits with a doctor or other health providers?

□ Yes □ No

5. [If “yes” to question #4] Did you have difficulty with making an appointment?

□ Yes □ No

6. [If “yes” to question #4] Were your telemedicine visits conducted through _________?

□ Telephone call

□ Video conference (e.g., Zoom, FaceTime, Skype, Webex, MS Teams)

□ Both

7. Were your telemedicine visits related to _________ ? Select all that apply.

□ Treatment consultation

□ Management of treatment side effects and/or cancer symptoms

□ Review of laboratory, screening, and/or pathology results

□ Cancer genetic counseling

□ Informed consent for a cancer clinical trial

□ Cancer clinical trial follow-up visits

□ Other, please specify: __________

8. [If “Management of treatment side effects and/or cancer symptoms” in question #7] What were your treatment side effects and/or symptoms discussed during your visits? Select all that apply.

□ Hot flashes

□ Chemotherapy-induced neuropathy (numbness or tingling in your hands/feet)

□ Nausea and/or vomiting

□ Pain related to cancer treatment (join pain, bone pain, etc.)

□ Pain from your cancer (bone pain, abdominal pain, etc.)

□ Depressive symptoms or mood changes

□ Tiredness or fatigue

□ Anxiety or stress

□ Hair loss

□ Lymphedema

□ Insomnia or sleep problems

9. How satisfied were you with your telephone call with doctors or other health care providers?

□ Not at all □ A Little □ Somewhat □ Very satisfied □ Extremely satisfied

10. How satisfied were you with your video conference with doctors or other health care providers?

□ Not at all □ A Little □ Somewhat □ Very satisfied □ Extremely satisfied

11. How satisfied were you with your in-person visit experience?

□ Not at all □ A Little □ Somewhat □ Very satisfied □ Extremely satisfied

12. How likely are you to continue using telemedicine?

□ Very unlikely □ Unlikely □ Neutral □ Likely □ Very likely

13. What were the challenge(s) or concern(s) when using telemedicine? Select that all apply.

□ Technology difficulty or lack of comfort with technology

□ Lack of an electronic device such as desktop, laptop, smartphone, and iPad

□ Lack of high-speed internet or slow in internet connection at home

□ Language barriers

□ Compromising the patient-provider communication

□ Compromising the patient-provider relationship

□ Not being offered at the clinic or by a provider/doctor

□ Cost

□ Not being covered by health insurance

□ Other, please specify: __________________________

□ None
