# Supplementary material for: Assessing the Relationship Between Neighborhood Socioeconomic Disadvantage and Telemedicine Use Among Patients With Breast Cancer and Examining Differential Provisions of Oncology Services Between Telehealth and In-Person Visits: Quantitative Study
Source: JMIR Cancer. 2024 Jul 18;10:e55438. doi: 10.2196/55438 (PMC11294759; doi:10.2196/55438)
Supplement: Multimedia Appendix 2 [file cancer_v10i1e55438_app2.docx]

**Supplemental Materials**

**Table S1.** Association between neighborhood socioeconomic disadvantage and telemedicine use in breast cancer patients

**Table S2.** Association between neighborhood socioeconomic disadvantage and telemedicine use (video conference vs. telephone) in breast cancer patients

**Table S3.** Percentages of oncology services among breast cancer patients by modality of telemedicine

**Table S4** Percentages of cancer symptoms and treatment side effects discussed during virtual visits reported by breast cancer patients by telemedicine modality

**Table S5.** Percentages of satisfaction with telephone call or videoconferencing by oncology services in breast cancer patients**.** Satisfaction with telemedicine visits and likelihood of future use reported by breast cancer patients.

**Table S6.** Percentages of satisfaction with virtual visits by oncology services in breast cancer patients

**Table S1.** Association between neighborhood socioeconomic disadvantage and telemedicine use in breast cancer patients

|  | Model 1 | Model 2 | Model 3 |
| --- | --- | --- | --- |
| Variable | AOR ^c^ (95% CI) | AOR ^d^ (95% CI) | AOR ^e^ (95% CI) |
| **Area Deprivation Index (continuous)** **^a,b^** | **0.93 (0.87-0.99) *** | **0.89 (0.82-0.96) ^†^** | **0.89 (0.82-0.97) ^†^** |
| **Distance from residence to hospital ^b^** | 1.04 (0.99-1.10) | 1.03 (0.97-1.09) | 1.02 (0.96-1.09) |
| **Age at survey (years)** |  |  |  |
| <45 | 1.0 (reference) | 1.0 (reference) | 1.0 (reference) |
| 45-54 | **0.55 (0.33-0.94) *** | **0.53 (0.29-0.97) *** | **0.49 (0.27-0.91) *** |
| 55-64 | **0.57 (0.35-0.93) *** | 0.64 (0.37-1.11) | 0.63 (0.36-1.12) |
| ≥65 | 0.65 (0.39-1.09) | 0.62 (0.34-1.13) | 0.63 (0.34-1.18) |
| **Race/Ethnicity** |  |  |  |
| White | 1.0 (reference) | 1.0 (reference) | 1.0 (reference) |
| Black | **1.86 (1.21-2.86) ^†^** | **2.50 (1.48-4.20) ^†^** | **2.38 (1.41-4.00) ^†^** |
| Asian | 0.55 90.26-1.17) | 0.50 (0.20-1.22) | 0.50 (0.20-1.23) |
| Hispanic | **2.12 (1.02-4.41) *** | **2.85 (1.17-6.91) *** | **2.65 (1.07-6.58) *** |
| **Highest level of education** |  |  |  |
| High school/GED or less | 1.0 (reference) | 1.0 (reference) | 1.0 (reference) |
| Associate’s degree, or some college | **2.66 (1.47-4.81) ^†^** | **2.76 (1.40-5.44) ^†^** | **2.67 (1.33-5.35) ^†^** |
| Bachelor’s degree | **2.43 (1.35-4.38) ^†^** | **2.61 (1.33-5.10) ^†^** | **2.75 (1.38-5.48) ^†^** |
| Graduate or professional degree | **2.46 (1.39-4.38) ^†^** | **2.55 (1.32-4.93) ^†^** | **2.57 (1.31-5.04) ^†^** |
| **Duration from cancer diagnosis to survey (years)** |  |  |  |
| ≤3 | 1.0 (reference) | 1.0 (reference) | 1.0 (reference) |
| 4-6 | **0.63 (0.41-0.96) *** | 0.67 (0.42-1.08) | 0.75 (0.46-1.21) |
| ≥7 | 0.67 (0.45-1.01) | **0.60 (0.38-0.96) *** | 0.65 (0.40-1.05) |
| **Marital status** |  |  |  |
| Married | 1.0 (reference) | 1.0 (reference) | 1.0 (reference) |
| Single or not married | 0.95 (0.64-1.42) | 0.95 (0.60-1.50) | 0.99 (0.62-1.58) |
| Divorced, separated, or widowed | 1.06 (0.66-1.70) | 1.27 (0.73-2.21) | 1.22 (0.69-2.17) |
| **Type of health insurance** |  |  |  |
| Private | 1.0 (reference) | 1.0 (reference) | 1.0 (reference) |
| Medicaid | 1.29 (0.62-2.68) | 0.87 (0.37-2.04) | 0.77 (0.32-1.89) |
| Medicare | 0.79 (0.51-1.22) | 0.89 (0.53-1.50) | 0.85 (0.50-1.44) |
| **Charlson comorbidity index** |  |  |  |
| 0 | 1.0 (reference) | 1.0 (reference) | 1.0 (reference) |
| 1 | 1.72 (0.96-3.08) | 1.29 (0.66-2.50) | 1.37 (0.70-2.67) |
| ≥2 | 1.01 (0.56-1.84) | 0.77 (0.35-1.70) | 0.79 (0.36-1.75) |
| **Histologic type** |  |  |  |
| Ductal |  | 1.0 (reference) | 1.0 (reference) |
| Lobular |  | 0.92 (0.53-1.60) | 0.84 (0.47-1.50) |
| Ductal and lobular |  | 0.55 (0.26-1.17) | 0.57 (0.26-1.21) |
| Other |  | 1.99 (0.81-4.85) | 2.02 (0.82-4.98) |
| **Molecular subtype** |  |  |  |
| HR+/HER2- |  | 1.0 (reference) | 1.0 (reference) |
| HR+/HER+ |  | 0.75 (0.43-1.31) | 0.69 (0.38-1.24) |
| HR-/HER2+ |  | 0.73 (0.33-1.60) | 0.52 (0.20-1.38) |
| TNBC |  | 0.84 (0.49-1.43) | 0.58 (0.26-1.29) |
| **Tumor grade** |  |  |  |
| 1 |  | 1.0 (reference) | 1.0 (reference) |
| 2 |  | 0.73 (0.44-1.21) | 0.77 (0.45-1.31) |
| 3 |  | 0.75 (0.42-1.34) | 0.79 (0.42-1.48) |
| **Receipt of chemotherapy** |  |  |  |
| No |  |  | 1.0 (reference) |
| Yes |  |  | 1.01 (0.61-1.67) |
| **Receipt of hormone therapy** |  |  |  |
| No |  |  | 1.0 (reference) |
| Yes |  |  | 0.72 (0.38-1.40) |
| **Receipt of radiation therapy** |  |  |  |
| No |  |  | 1.0 (reference) |
| Yes |  |  | 0.95 (0.61-1.49) |
| **Type of surgery received** |  |  |  |
| None |  |  | 1.0 (reference) |
| Lumpectomy |  |  | 0.27 (0.05-1.45) |
| Mastectomy |  |  | 0.29 (0.06-1.48) |
| Bilateral mastectomy |  |  | 0.25 (0.05-1.41) |

Abbreviations: SD, standard deviation; GED, general educational development; AOR, adjusted odds ratio; CI, confidence interval;

^a^ The Area Deprivation Index (national ranking percentile), a composite measure consisting domains of income, education, employment, and housing quality, that ranks neighborhoods by socioeconomic disadvantage at the national level. It is scored from 1 to 100, with higher scores representing greater neighborhood socioeconomic deprivation.

^b^ Odds ratios were per 10-unit increase.

^c^ Additionally adjusted for marital status, health insurance, and Charlson comorbidity index.

^d^ Additionally adjusted for marital status, health insurance, Charlson comorbidity index, histologic type, stage, molecular subtype, and tumor grade.

^e^ Additionally adjusted for marital status, type of health insurance, Charlson comorbidity index, histologic type, stage, molecular subtype, tumor grade, receipt of chemotherapy, of hormone therapy, or of radiotherapy, and type of surgery.

* *p*<0.05; † *p*<0.01.

**Table S2.** Association between neighborhood socioeconomic disadvantage and telemedicine use (video conference vs. telephone) in breast cancer patients

|  | Model 1 | Model 2 | Model 3 |
| --- | --- | --- | --- |
| Variable | AOR ^c^ (95% CI) | AOR ^d^ (95% CI) | AOR ^e^ (95% CI) |
| **Area Deprivation Index (continuous)** **^a,b^** | 0.91 (0.79-1.04) | 0.86 (0.72-1.04) | 0.88 (0.73-1.07) |
| **Distance from residence to hospital ^b^** | 1.02 (0.92-1.14) | 1.09 (0.93-1.28) | 1.09 (0.93-1.28) |
| **Age at survey (years)** |  |  |  |
| <45 | 1.0 (reference) | 1.0 (reference) | 1.0 (reference) |
| 45-54 | 1.98 (0.58-6.70) | 1.23 (0.29-5.30) | 1.15 (0.24-5.63) |
| 55-64 | 0.57 (0.21-1.56) | 0.58 (0.16-2.15) | 0.43 (0.10-1.91) |
| ≥65 | 0.63 (0.21-1.87) | 0.49 (0.11-2.10) | 0.45 (0.09-2.26) |
| **Race/Ethnicity** |  |  |  |
| White | 1.0 (reference) | 1.0 (reference) | 1.0 (reference) |
| Black | 1.00 (0.44-2.25) | 2.63 (0.75-9.24) | 2.64 (0.73-9.51) |
| Asian | 0.87 (0.15-5.11) | 1.68 (0.11-25.91) | 1.11 (0.07-16.81) |
| Hispanic | 0.69 (0.19-2.48) | 0.59 (0.12-2.85) | 0.35 (0.06-2.14) |
| **Highest level of education** |  |  |  |
| High school/GED or less | 1.0 (reference) | 1.0 (reference) | 1.0 (reference) |
| Associate’s degree, or some college | 1.65 (0.44-6.19) | 3.01 (0.58-15.69) | 2.71 (0.46-15.78) |
| Bachelor’s degree | 2.42 (0.67-8.79) | 4.81 (0.98-23.61) | 4.36 (0.77-24.68) |
| Graduate or professional degree | 2.47 (0.69-8.87) | **6.67 (1.34-33.26) *** | **5.78 (1.03-32.55) *** |
| **Duration from cancer diagnosis to survey (years)** |  |  |  |
| ≤3 | 1.0 (reference) | 1.0 (reference) | 1.0 (reference) |
| 4-6 | 1.62 (0.70-3.76) | 2.44 (0.86-6.91) | 1.73 (0.56-5.37) |
| ≥7 | 1.37 (0.64-2.97) | 1.40 (0.54-3.66) | 1.02 (0.36-2.93) |
| **Marital status** |  |  |  |
| Married | 1.0 (reference) | 1.0 (reference) | 1.0 (reference) |
| Single or not married | 1.03 (0.47-6.19) | 1.35 (0.49-3.73) | 1.21 (0.40-3.65) |
| Divorced, separated, or widowed | 1.51 (0.57-4.01) | 1.83 (0.51-6.49) | 1.81 (0.44-7.47) |
| **Type of health insurance** |  |  |  |
| Private | 1.0 (reference) | 1.0 (reference) | 1.0 (reference) |
| Medicaid | 0.55 (0.14-2.15) | 1.09 (0.14-8.45) | 0.83 (0.09-7.48) |
| Medicare | **0.36 (0.15-0.86) *** | **0.29 (0.09-0.92) *** | **0.26 (0.07-0.91) *** |
| **Charlson comorbidity index** |  |  |  |
| 0 | 1.0 (reference) | 1.0 (reference) | 1.0 (reference) |
| 1 | 1.70 (0.50-5.83) | 1.88 (0.35-10.18) | 1.36 (0.22-8.31) |
| ≥2 | 1.56 (0.38-6.36) | 0.69 (0.10-4.70) | 0.52 (0.07-3.68) |
| **Histologic type** |  |  |  |
| Ductal |  | 1.0 (reference) | 1.0 (reference) |
| Lobular |  | 0.68 (0.18-2.61) | 0.31 (0.07-1.37) |
| Ductal and lobular |  | 0.50 (0.09-2.72) | 0.53 (0.08-3.35) |
| Other |  | 0.24 (0.04-1.45) | 0.10 (0.01-0.96) |
| AJCC stage |  |  |  |
| I |  | 1.0 (reference) | 1.0 (reference) |
| II |  | 0.55 (0.25-1.22) | 0.70 (0.27-1.82) |
| III-IV |  | 0.72 (0.20-2.66) | 0.98 (0.21-4.53) |
| **Molecular subtype** |  |  |  |
| HR+/HER2- |  | 1.0 (reference) | 1.0 (reference) |
| HR+/HER+ |  | 14.62 (1.51-141.50) | –– |
| HR-/HER2+ |  | 1.29 (0.25-6.58) | 0.85 (0.10-7.25) |
| TNBC |  | 1.80 (0.56-5.78) | 2.54 (0.43-15.10) |
| **Tumor grade** |  |  |  |
| 1 |  | 1.0 (reference) | 1.0 (reference) |
| 2 |  | 0.95 (0.29-3.08) | 1.18 (0.31-4.42) |
| 3 |  | 0.52 (0.15-1.74) | 0.60 (0.15-2.38) |
| **Receipt of chemotherapy** |  |  |  |
| No |  |  | 1.0 (reference) |
| Yes |  |  | 0.75 (0.25-2.05) |
| **Receipt of hormone therapy** |  |  |  |
| No |  |  | 1.0 (reference) |
| Yes |  |  | 1.09 (0.24-4.94) |
| **Receipt of radiation therapy** |  |  |  |
| No |  |  | 1.0 (reference) |
| Yes |  |  | 0.24 (0.07-0.80) |
| **Type of surgery received** |  |  |  |
| None |  |  | 1.0 (reference) |
| Lumpectomy |  |  | 1.55 (0.04-59.64) |
| Mastectomy |  |  | 1.36 (0.03-55.97) |
| Bilateral mastectomy |  |  | 0.38 (0.01-18.74) |

Abbreviations: SD, standard deviation; GED, general educational development; AOR, adjusted odds ratio; CI, confidence interval;

^a^ The Area Deprivation Index (national ranking percentile), a composite measure consisting domains of income, education, employment, and housing quality, that ranks neighborhoods by socioeconomic disadvantage at the national level. It is scored from 1 to 100, with higher scores representing greater neighborhood socioeconomic deprivation.

^b^ Odds ratios were per 10-unit increase.

^c^ Additionally adjusted for marital status, health insurance, and Charlson comorbidity index.

^d^ Additionally adjusted for marital status, health insurance, Charlson comorbidity index, histologic type, stage, molecular subtype, and tumor grade.

^e^ Additionally adjusted for marital status, type of health insurance, Charlson comorbidity index, histologic type, stage, molecular subtype, tumor grade, receipt of chemotherapy, of hormone therapy, or of radiotherapy, and type of surgery.

* p<0.05.

**Table S3.** Percentages of oncology services among breast cancer patients by modality of telemedicine

|  | **Modality of telemedicine**  (n=409) | | |  |
| --- | --- | --- | --- | --- |
| **Variable** | **Telephone call**  (n=93 [22.7%]),  n (col %) | **Video Conference** (n=266 [65.0%]),  n (col %) | **Both**  (n=50 [12.3%]),  n (col %) | ***P* value ^a^** |
| **Treatment consultation** |  |  |  |  |
| No | 76 (81.7) | 178 (66.9) | 27 (54.0) | 0.002 |
| Yes | 17 (18.3) | 88 (33.1) | 23 (46.0) |  |
| **Review of laboratory, screening and/or pathology results** |  |  |  |  |
| No | 71 (76.3) | 214 (80.5) | 32 (64.0) | 0.036 |
| Yes | 22 (23.7) | 52 (19.6) | 18 (36.0) |  |
| **Management of treatment side effects or cancer symptoms** |  |  |  |  |
| No | 83 (89.3) | 228 (85.7) | 42 (84.0) | 0.611 |
| Yes | 10 (10.8) | 38 (14.3) | 8 (16.0) |  |
| **Cancer genetic counseling** |  |  |  |  |
| No | 89 (95.7) | 256 (96.2) | 46 (92.0) | 0.381 |
| Yes | 4 (4.3) | 10 (3.8) | 4 (8.0) |  |
| **Cancer clinical trial follow-up visits** |  |  |  |  |
| No | 93 (100.0) | 253 (95.1) | 49 (98.0) | 0.060 |
| Yes | 0 | 13 (4.9) | 1 (2.0) |  |

^a^ *P* values were calculated using Pearson’s *X^2^* or Fisher’s exact tests.

**Table S4.** Percentages of cancer symptoms and treatment side effects discussed during virtual visits reported by breast cancer patients by telemedicine modality

|  | **Modality of telemedicine**  (n=56) | | |  |
| --- | --- | --- | --- | --- |
| **Variable** | **Telephone call** (n=10 [17.9%]), n (col %) | **Video Conference**  (n=38 [67.9%]), n (col %) | **Both**  (n=8 [14.3%]), n (col %) | ***P* value ^a^** |
| **Hot flashes** |  |  |  |  |
| No | 8 (80.0) | 23 (60.5) | 6 (75.0) | 0.577 |
| Yes | 2 (20.0) | 15 (39.5) | 2 (25.0) |  |
| **Chemotherapy-induced neuropathy** |  |  |  |  |
| No | 9 (90.0) | 31 (81.6) | 5 (62.5) | 0.403 |
| Yes | 1 (10.0) | 7 (18.4) | 3 (37.5) |  |
| **Nausea and/or vomiting** |  |  |  |  |
| No | 8 (80.0) | 35 (92.1) | 8 (100.0) | 0.344 |
| Yes | 2 (20.0) | 3 (7.9) | 0 |  |
| **Pain related to cancer treatment** |  |  |  |  |
| No | 7 (70.0) | 24 (63.2) | 4 (50.0) | 0.771 |
| Yes | 3 (30.0) | 14 (36.8) | 4 (50.0) |  |
| **Pain from cancer** |  |  |  |  |
| No | 9 (90.0) | 37 (97.4) | 7 (87.5) | 0.239 |
| Yes | 1 (10.0) | 1 (2.6) | 1 (12.5) |  |
| **Depressive symptoms or mood changes** |  |  |  |  |
| No | 8 (80.0) | 22 (57.9) | 5 (62.5) | 0.489 |
| Yes | 2 (20.0) | 16 (42.1) | 3 (37.5) |  |
| **Fatigue or tiredness** |  |  |  |  |
| No | 7 (70.0) | 23 (60.5) | 3 (37.5) | 0.392 |
| Yes | 3 (30.0) | 15 (39.5) | 5 (62.5) |  |
| **Anxiety or stress** |  |  |  |  |
| No | 8 (80.0) | 24 (63.2) | 4 (50.0) | 0.442 |
| Yes | 2 (20.0) | 14 (36.8) | 4 (50.0) |  |
| **Hair loss** |  |  |  |  |
| No | 9 (90.0) | 33 (86.8) | 5 (62.5) | 0.205 |
| Yes | 1 (10.0) | 5 (13.2) | 3 (37.5) |  |
| **Lymphedema** |  |  |  |  |
| No | 8 (80.0) | 30 (79.0) | 7 (87.5) | 1.000 |
| Yes | 2 (20.0) | 8 (21.1) | 1 (12.5) |  |
| **Insomnia or sleep problems** |  |  |  |  |
| No | 8 (80.0) | 27 (71.1) | 7 (87.5) | 0.725 |
| Yes | 2 (20.0) | 11 (29.0) | 1 (12.5) |  |

^a^ *P* values were calculated using Fisher’s exact tests.

**Table S5.** Percentages of satisfaction with telephone call or videoconferencing by oncology services in breast cancer patients**.** Satisfaction with telemedicine visits and likelihood of future use reported by breast cancer patients.

| Variable | Overall  n (%) |
| --- | --- |
| **Did you have difficulty with making a telemedicine appointment?** (n=415) |  |
| No | 398 (95.9) |
| Yes | 17 (4.1) |
| **Did you have difficulty with making an in-person appointment?** (n=1,067) |  |
| No | 994 (93.2) |
| Yes | 73 (6.8) |
| **How satisfied were you with your telephone call with doctors or other health care providers?** (n=141) |  |
| Not at all | 2 (1.4) |
| A little | 7 (5.0) |
| Somewhat | 28 (19.9) |
| Very | 62 (44.0) |
| Extremely | 42 (29.8) |
| **How satisfied were you with your video conference with doctors or other health care providers?** (n=315) |  |
| Not at all | 8 (2.5) |
| A little | 6 (1.9) |
| Somewhat | 56 (17.8) |
| Very | 157 (49.8) |
| Extremely | 88 (27.9) |
| **How satisfied were you with your telemedicine (telephone call or video conference with providers) visits with doctors or other health care providers?** (n=407) |  |
| Not at all | 8 (2.0) |
| A little | 9 (2.2) |
| Somewhat | 74 (18.2) |
| Very | 195 (47.9) |
| Extremely | 121 (29.7) |
| **How satisfied were you with your in-person visit experience?** (n=1,053) |  |
| Not at all | 11 (1.0) |
| A little | 9 (0.9) |
| Somewhat | 108 (10.3) |
| Very | 495 (47.0) |
| Extremely | 430 (40.8) |
| **How likely are you to continue using telemedicine?** (n=411) |  |
| Very unlikely | 16 (3.9) |
| Unlikely | 31 (7.5) |
| Neutral | 110 (26.8) |
| Likely | 121 (29.4) |
| Very likely | 133 (32.4) |

**Table S6.** Percentages of satisfaction with virtual visits by oncology services in breast cancer patients

|  | **How satisfied were you with your virtual visits**  **(telephone call and/or video conference with providers) (n=407)** | | | | |  |
| --- | --- | --- | --- | --- | --- | --- |
| **Were your telemedicine visits related to the following oncology services?** | **Not at all**  (n=8 [2.0%]),  n (row %) | **A little**  (n=9 [2.2%]),  n (row %) | **Somewhat** (n= [%]),  n (row %) | **Very**  (n=195 [47.9%]),  n (row %) | **Extremely**  (n=121 [29.7%]),  n (row %) | ***P* value ^a^** |
| **Treatment consultation** |  |  |  |  |  |  |
| No | 5 (1.8) | 7 (2.5) | 52 (18.6) | 134 (48.0) | 81 (29.0) | 0.600 |
| Yes | 3 (2.3) | 2 (1.6) | 22 (17.2) | 61 (47.7) | 40 (31.3) |  |
| **Review of laboratory, screening and/or pathology results** |  |  |  |  |  |  |
| No | 7 (2.2) | 6 (1.9) | 63 (20.0) | 144 (45.7) | 95 (30.2) | 0.560 |
| Yes | 1 (1.1) | 3 (3.3) | 11 (12.0) | 51 (55.4) | 26 (28.3) |  |
| **Management of treatment side effects or cancer symptoms** |  |  |  |  |  |  |
| No | 7 (2.0) | 9 (2.6) | 65 (18.5) | 165 (47.0) | 105 (29.9) | 0.699 |
| Yes | 1 (1.8) | 0 | 9 (16.1) | 30 (53.6) | 16 (28.6) |  |
| **Cancer genetic counselling** |  |  |  |  |  |  |
| No | 7 (1.8) | 9 (2.3) | 70 (18.0) | 186 (47.8) | 117 (30.1) | 0.425 |
| Yes | 1 (5.6) | 0 | 4 (22.2) | 9 (50.0) | 4 (22.2) |  |
| **Cancer clinical trial follow-up visits** |  |  |  |  |  |  |
| No | 8 (2.0) | 9 (2.3) | 72 (18.3) | 184 (46.8) | 120 (30.5) | 0.423 |
| Yes | 0 | 0 | 2 (14.3) | 11 (78.6) | 1 (7.1) |  |

^a^ *P* values were calculated using Wilcoxon rank-sum tests.
